# A Consideration of the Causes and Prevention of Primary Pneumonias and Pneumonias Complicating Measles in the United States Army

**Published:** 1918-11

**Authors:** 


**A Consideration of the Causes and Prevention of Primary Pneumonias and Pneumonias Complicating Measles in the United States Army**. By Captain WALLACE A. PRATT., *Military Surgeon*, June, 1918.

The prophylactic measures against pneumonia should first of all endeavor to strengthen the resistance of the organism against pneumonic bacilli. These are probably of four different types, but the streptococcus also plays a prominent part in the so-called pneumonias.

The author considers that a certain number of factors in the present methods of living in quarters inside the camps break down considerably the resistance of men against infection. These are principally : *inefficient bedding, improper ventilation, superheated rooms.*

1) The regulation army blanket is woefully insufficient. It would take from 3 to 4 of them to keep a man warm even in a mild climate. Mattress or under-blankets are also insufficiently protective. Owing to this, the men do not rest well, and when they get up in the morning they do not possess their full resistance against infective bacteria.

2) Whatever orders are given, windows are generally closed at night in the barracks because of the cold; the cots are close together, and the air is contaminated by breathing.

3) During evening hours, the stoves are heating briskly. The air is over-heated to such an extent that the upper air passages of a large majority of men are dry, thus allowing of an easy invasion by the bacilli. The following measures should be prescribed, and would probably prove of some use to keep pneumonia from camps :

— Sufficient rest in bed should be secured by suppressing evening leaves during winter time, and the men should be provided with thicker or differently woven blankets.

— Better arrangements should be provided for the cots, with a good mattress, or straw tick.

— Thermometers should be placed in convenient places in all barracks, and temperature should never exceed 70° F.

— Water pans should be placed over all stoves, and special men detailed to keep water in them constantly.

*Transportation of Troops on Trains:* This is generally done under most unhygienic conditions. The men are seated sometimes for several days, with no exercises at all. Thus the resistance becomes very low, and the possibilities for infection most marked.

*Preventive Measures :* The care of the teeth should be imperative four times daily. In each car a reliable man should be detailed to open windows for five minutes every hour during day-time. Temperature in the cars should never exceed 68° F. Setting up exercises, and especially leg exercises, should be required every 3 hours throughout the day. At division points, all men should march briskly for 20 minutes.

*Pneumonia Complicating Measles :* It is now realized that patients suffering with measles take up the pneumococci readily, and undoubtedly also many other pathogenic organisms. To prevent these complications, it is advisable to give the patients abundant fresh air, and put them in alternating beds, head and feet. Teeth should be cleaned four times a day; a mouth gargle, such as Dobell's sol., or saturated boric acide sol., used twice daily; and anterior nares and throat should be sprayed with a mild antiseptic spray, such as liquor antisepticus.

